# Prognostic Factors and Clinical Outcomes of Surgical Treatment of Major Thoracic Trauma

**DOI:** 10.3390/healthcare12111147

**Published:** 2024-06-05

**Authors:** Maria Chiara Sibilia, Federica Danuzzo, Francesca Spinelli, Enrico Mario Cassina, Lidia Libretti, Emanuele Pirondini, Federico Raveglia, Antonio Tuoro, Luca Bertolaccini, Stefano Isgro’, Stefano Perrone, Stefania Rizzo, Francesco Petrella

**Affiliations:** 1 Department of Thoracic Surgery, Fondazione IRCCS San Gerardo dei Tintori, 20900 Monza, Italy; maria.sibilia@unimi.it (M.C.S.); federica.danuzzo@unimi.it (F.D.); francesca.spinelli@unimi.it (F.S.); enricomario.cassina@irccs-sangerardo.it (E.M.C.); lidia.libretti@irccs-sangerardo.it (L.L.); emanuele.pirondini@irccs-sangerardo.it (E.P.); federico.raveglia@irccs-sangerardo.it (F.R.); antonio.tuoro@irccs-sangerardo.it (A.T.); 2 Department of Thoracic Surgery, IEO, European Institute of Oncology IRCCS, Via Ripamonti 234, 20141 Milan, Italy; luca.bertolaccini@ieo.it; 3 Department of Emergency and Intensive Care, Fondazione IRCCS San Gerardo dei Tintori, 20900 Monza, Italy; stefano.isgro@irccs-sangerardo.it; 4 Department of Surgery, Fondazione IRCCS San Gerardo dei Tintori, 20900 Monza, Italy; stefano.perrone@irccs-sangerardo.it; 5 Service of Radiology, Imaging Institute of Southern Switzerland (IIMSI), EOC Via Tesserete 46, 6900 Lugano, Switzerland; stefaniamariarita.rizzo@eoc.ch; 6 Facoltà di Scienze Biomediche, Università della Svizzera Italiana (USI), Via Buffi 13, 6900 Lugano, Switzerland

**Keywords:** trauma, chest wall, lung, Abbreviated Injury Scale (AIS), Injury Severity Score (ISS), accident and emergency

## Abstract

Background: Major thoracic trauma represents a life-threatening condition, requiring a prompt multidisciplinary approach and appropriate pathways for effective recovery. While acute morbidity and mortality are well-known outcomes in thoracic-traumatized patients, long-term quality of life in patients surviving surgical treatment has not been widely investigated before. Methods: Between November 2016 and November 2023, thirty-two consecutive patients were operated on because of thoracic trauma. Age, sex, comorbidities, location and extent of thoracic trauma, Injury Severity Score (ISS), Abbreviated Injury Scale (AIS), Organ Injury Scale (OIS), intra and extrathoracic organ involvement, mechanism of injury, type of surgical procedure, postoperative complications, ICU and total length of stay, immediate clinical outcomes and long-term quality of life—by using the EQ-5D-3L scale and Numeric Rate Pain Score (NPRS)—were collected for each patient Results: Results indicated no significant difference in EQOL.5D3L among patients with thoracic trauma based on AIS (*p* = 0.55), but a significant difference was observed in relation to ISS (*p* = 0.000011). Conclusions: ISS is correlated with the EQOL.5D3L questionnaire on long-term quality of life, representing the best prognostic factor—in terms of long-term quality of life—in patients surviving major thoracic trauma surgical treatment.

## 1. Introduction

Approximately 20–25% of deaths in polytrauma patients are due to chest injury [1]. The most common diagnostic imaging modality in polytrauma patients is computed tomography [2], although other modalities can be used during the follow-up and for prognostication [3,4,5]. The mortality rate in patients receiving blunt chest trauma is reported to be 60% both in Europe and the United States, representing a life-threatening condition because of airway damage and cardiopulmonary impairment [6]. Rib fractures occur in 10% of injured patients, significantly incrementing morbidity and mortality of traumatic events. In fact, respiratory distress and respiratory failure can happen because of impaired rib cage mechanics due to fractures themselves and fracture-associated pain [7]. Moreover, concurrent lung contusions—which are often observed after thoracic trauma—further contribute to worsen the hypoxia related to chest wall trauma. Patients presenting with major thoracic trauma, therefore, usually require oro-tracheal intubation for prolonged mechanical ventilation, thus increasing the risk of severe ventilator-dependent pneumonia and prolonged intensive care unit (ICU) length of stay [8]. Older age, a higher number of fractured ribs as well as bilateral rib fractures have been shown to increase post-traumatic morbidity and mortality; on the other hand, younger patients usually present an increased number of fractured ribs compared to older patients, because of greater kinetic injury while older patients’ fractures are more often related to decreased bone density rather than to major thoracic trauma with other organ involvement [9].

Other variables influencing the post-traumatic course are pulmonary contusions, hemothorax, pneumothorax, combined hemo-pneumothorax and flail chest. The basic principles for managing thoracic trauma patients are: early mobilization, whenever feasible in the context of polytrauma, pulmonary endoscopic toilette in the case of sputum retention and proper pain management by epidural analgesia or patient-controlled analgesia. Pain contributes to worsening post-traumatic morbidity; optimal analgesic treatment in the first hours, in combination with respiratory physiotherapy and early mobilization, is an essential component in the treatment of closed chest trauma and improves its outcome [10]. Optimal pain management is pivotal in the case of rib and sternal fractures to allow deep breathing, improved respiratory excursion and better respiratory function, which are the basis for an effective physiotherapeutic approach. In the case of more complex post-traumatic injuries, both mechanical ventilation and early surgical repair of sternal and rib fractures represent the best approaches to reduce respiratory complications [9]. The analgesic strategy includes multimodal analgesia (NSAIDs, paracetamol, gabapentin/pregabalin and opioids given together) alone or in combination with locoregional analgesia techniques such as epidural and fascial plane blocks (paravertebral, ESPBs, serratus block) [10,11,12,13].

Although it is well known that earlier interventions in post-traumatic scenarios provide the greatest benefit for the patient, an effective method to predict the clinical courses of these patients is not yet available. The aim of the present paper is to assess prognostic factors and to foresee clinical outcomes of surgically treated patients after major thoracic trauma.

## 2. Materials and Methods

This was a single-center, retrospective, observational study conducted in accordance with the Declaration of Helsinki as revised in 2013 [14]. Being a study conducted in an emergency setting, written informed consent to undergo the procedure and for the use of clinical and imaging data for scientific or educational purposes, or both, was obtained when patients were able to clearly express their will.

This study was approved by the medical ethics committee of our Institution (ID 4594).

Between November 2016 and November 2023, thirty-two consecutive patients were operated on at the Fondazione IRCCS San Gerardo dei Tintori—which is a referral center for major trauma—because of thoracic trauma. Thoracic trauma was defined as any form of physical injury to the chest including the ribs, sternum, heart, lungs and any other organ within the chest, with or without extrathoracic concurrent trauma (polytrauma). Among them, 3 were lost to follow up because of logistic reasons (foreign patients living abroad) and one was still admitted at the time this article was written. Data were therefore extracted from 28 operated patients.

Age, sex, comorbidities, location and extent of thoracic trauma, Injury Severity Score (ISS), Abbreviated Injury Scale (AIS), Organ Injury Scale (OIS), intra- and extrathoracic organ involvement, mechanism of injury, type of surgical procedure, postoperative complications, ICU and total length of stay, immediate clinical outcomes and long-term quality of life—by using the EQ-5D-3L scale and Numeric Rate Pain Score (NPRS)—were collected for each patient.

The EQ-5D-3L scale quality of life assessment is one of the most effective questionnaires in investigating the long-term impact of post-traumatic sequelae in surviving patients: it consists of a descriptive system investigating quality of life by exploring five basic health and functional dimensions: mobility, self-care, usual activities, pain discomfort and anxiety/depression. Each aspect is further subclassified into three levels of perceived problems indicated in the answers: patients are asked if they have no problems = 1, some problems = 2 or extreme problems = 3. The answers provided for the five dimensions are then converted into a summary score, which indicates the overall utility.

The Abbreviated Injury Scale (AIS) groups trauma descriptors in body anatomical regions and assigns a numerical value from 1 to 6. The Injury Severity Score (ISS) is a numerical ordinal scale, ranging from 1 to 75, taking into consideration six anatomical regions and calculated as the sum of the squares of the highest AIS scores for the three most severely affected anatomical regions.

### Statistical Methods

Descriptive statistics were used to summarize data regarding demographic characteristics. Continuous variables were reported as median (range) and compared between groups using unpaired Student’s t-tests assuming equal variance. The normality of data distribution was assessed with the Kolmogorov–Smirnov test. Categorical variables were reported as absolute value and percentage; differences in their distribution between groups were analyzed using chi-square tests. To identify preoperative clinical risk factors of uneventful outcomes, univariable logistic regression analysis was performed on a set of variables deemed relevant a priori. Variables exhibiting a *p*-value ≤ 0.10 were included in the multivariable logistic regression. As a robustness check, a further analysis based on multivariable logistic regressions was implemented by computing standard errors corrected for cluster correlation. The incidence of specific complications and their correlation with preoperative factors was assessed by implementing Spearman tests. The impact of trauma on EuroQoL-5D-3L (EQ-5D-3L) scores at follow up (1–72 months) was assessed using Tobit regressions. Significance was defined as a *p*-value less than 0.05. RStudio (R version 4.2.1, Funny-Looking Kid) was utilized for data analyses. The standard, *EZR*, *irr*, and *rcmdr* packages were used for statistical analysis.

## 3. Results

Among the study population, 17 patients (60.7%) were male; the median age was 60.3 years (range: 24–80 years). A total of 15 patients (53.5%) had comorbidities: 7 patients (28.5%) had major comorbidities (ischemic heart disease, decompensated diabetes mellitus, epilepsy, renal failure), and 8 patients (25%) had minor comorbidities (dyslipidemia, hypertension, depressive syndrome). The median total length of stay (LOS) was 17 days (range: 2–86 days); the mean ICU LOS was 8 days (range: 0–43 days). The median time between admission and thoracic surgical procedure was 3 days (range: 0–15 days). The median number of fractured ribs was 9 (range: 2–20); 6 patients (21.4%) suffered from sternal fractures, 21 patients had flail chest (75%) and 18 patients (64.2%) suffered from bilateral thoracic trauma. The median number of repaired ribs was 3, range: 1–8. Sternal stabilization was necessary in 2 cases (7.1%). A total of 15 patients (53.5%) suffered from lung contusion; 12 patients (53.5%) suffered from pneumothorax alone while 3 patients (10.7%) presented pneumothorax associated with other intrathoracic injuries (diaphragm, vessels, hemo-pneumothorax); 2 patients (7.1%) disclosed only vascular involvement and one patient (3.5%) only diaphragm involvement.

Intrathoracic lesions—other than skeletal lesions—were reported in 19 cases: only lung in 12 cases; only vessels in 2 cases; lung + other in 2 cases; lung + vessel + diaphragm in 1 case; only diaphragm in 1 case; lung + vessel in 1 case.

**Figure 1:**
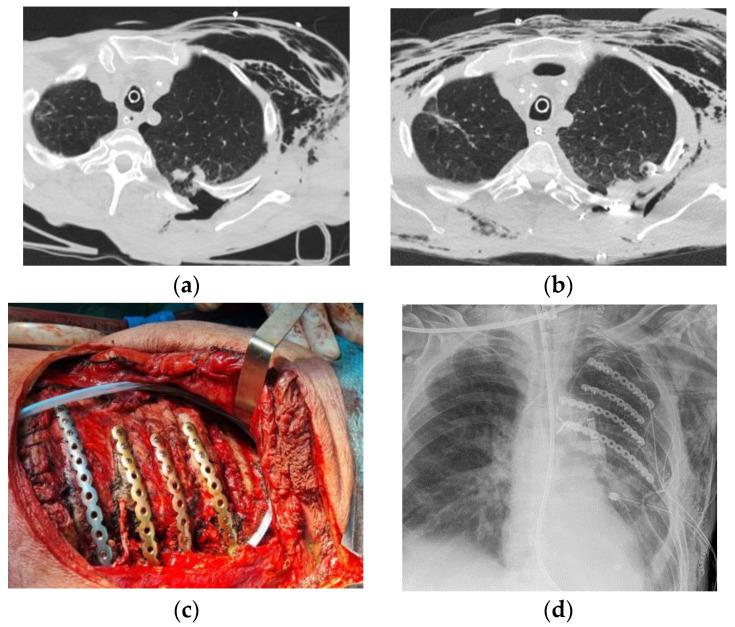
(**a**) Preoperative computed tomography disclosing rib fracture and penetrating lung injury. (**b**) Postoperative computed tomography disclosing rib stabilization by plate and screws. (**c**) Intraoperative view disclosing plates, screws and stitches. (**d**) Postoperative chest X rays.

A total of 21 patients (75%) had concurrent extrathoracic trauma which were combined with other forms of trauma in 7 patients (25%): orthopedic involvement was reported in 13 patients (46.4%), central nervous system trauma was observed in 6 patients (21.4%), visceral trauma was reported in 6 patients (21.4%), vascular injuries were observed in 4 patients (14.2%) while other regions were involved in 1 case (ocular trauma) (3.5%). With regard to the trauma dynamics, we observed: 7 cases (25%) vehicle vs. vehicle, 9 cases (32.1%) vehicle vs. non-vehicle, 5 cases (17.8%) falling from heights less than 2 m, 2 cases (7.1%) falling from more than 2 m and 5 cases (17.8%) involving other dynamics (e.g., vehicle vs. wall). We did not observe trauma from assault or penetrating objects. Four patients (14.2%) arrived at the hospital already intubated or were intubated upon arrival at the emergency room. Sixteen patients (57.1%) received chest drainage before surgery, and two patients (7.1%) required intercostal artery embolization. Patients were operated on by using screws and plates in 24 cases (85.7%), stitches in 1 case (3.5%) and a combined technique in 3 cases (10.7%). In addition to chest wall repair, a chest wall prosthesis was placed in 1 case (3.5%) (Figure 1). The median duration of the thoracic surgical procedure was 135 min (range: 60–315). In three cases, the thoracic surgical procedure was performed within a synchronous multidisciplinary approach involving general surgeons, orthopedic surgeons and neurosurgeons; a selective thoracic procedure duration in these cases was not recorded.

Perioperative blood transfusions were required in 16 patients (57.1%). Median Injury Severity Score (ISS) was 21 (range: 4–75); the median Abbreviated Injury Scale (AIS) was 3 (range: 2–4); the median Organ Injury Scale (OIS) was 2 (range: 1–5). Nine patients (32.1%) developed postoperative pneumonia; other complications were observed in five patients (17.8%) including cardiac arrythmia, ictus cerebri and chest wall hematoma. Two patients (7.1%) required re-operation for bleeding and chest wall hematoma. One patient (3.5%) died because of a concurrent major brain injury during the in-hospital stay. The median follow-up duration was 14 months (range: 1–72): we registered one death due to causes unrelated to the trauma; the median EQ-5D-3L scale quality of life assessment score was 0.857 (range: 0.027–1); the EQ-5D-3L scale quality of life results did not correlate with follow up duration. None of our patients reported the presence of dyspnea at rest, while eight patients (28.5%) reported exertional dyspnea. The median Numerical Rate Pain Score (NRPS) was 2 (range: 1–6) (Table 1).

Initially, Spearman Rank Order non-parametric tests were conducted to assess correlations. It was observed that EQOL.5D3L exhibited a significant negative correlation with pain (rho = −0.60), whereas other correlations showed negligible indices. Importantly, the Injury Severity Score (ISS) emerged as a predictor of prolonged intensive care unit (ICU) stay (rho = 0.66) and, consequently, overall hospitalization (rho = 0.69). Subsequently, a linear logistic regression analysis was performed to evaluate variables correlated with EQOL.5D3L. AIS has not demonstrated a significant impact, with an HR (hazard ratio) of 1.11, a 95% confidence interval ranging from 0.87 to 2.62, and a *p*-value of 0.55. On the other hand, ISS demonstrated a significant impact on the model, with an HR (hazard ratio) of 1.64, a 95% confidence interval ranging from 1.14 to 3.83, and a *p*-value of 0.034. Finally, the Log-Rank test was conducted to assess the quality of life by arbitrarily stratifying EQOL.5D3L into “good” (>0.50) and “bad” (<0.49) categories based on AIS (Abbreviated Injury Scale) and ISS. Results indicated no significant difference in EQOL.5D3L among patients with thoracic trauma based on AIS (*p* = 0.55), but a significant difference was observed in relation to ISS (*p* = 0.000011). In conclusion, ISS (rather than AIS) demonstrated a correlation with EQOL.5D3L and emerged as a significant prognostic factor for quality of life in patients with thoracic trauma.

## 4. Discussion

Rib fractures are frequently observed in blunt trauma patients, having been reported in approximately 20% of cases of thoracic trauma [15]. Due to the pain caused by rib fractures, patients are not able to effectively cough, thus inducing secretion retention and lobe or lung atelectasis culminating in severe pneumonia. This condition—often associated with coexisting lung contusions and hemo-pneumothorax—may lead to respiratory failure, representing a difficult-to-manage life-threatening condition [16]. When three or more consecutive ribs show fractures in at least two different sites of the costal arch, this is defined as flail chest, causing paradoxical chest physiology which severely interferes with spontaneous ventilation, worsening the prognosis and resulting in a mortality rate up to 30%. Patients surviving major thoracic trauma with rib and sternal involvement often suffer from chronic pain seriously affecting their long-term quality of life [17].

The classification of traumatic events—based on their gravity and category—is paramount in investigating their impact and determinants as well as in identifying the best therapeutic pathway. Since the early 1960s, several grading scales have been proposed to assess and categorize injury severity: the Abbreviated Injury Scale (AIS) was one of the first and is the most used, grouping trauma descriptors in body regions and assigning a numerical value from 1 to 6. In the case of polytrauma, the highest reported AIS is defined as Maximum AIS (MAIS) which—although considered as a good descriptor of the overall severity—is not linearly correlated with the probability of death [18,19]. To overcome this limitation, Baker et coll. described a derivative measure of the AIS, defined Injury severity score (ISS). The ISS is a numerical ordinal scale, ranging from 1 to 75, taking into consideration six anatomical regions and calculated as the sum of the squares of the highest AIS scores for the three most severely affected anatomical regions [20].

The EQ-5D-3L descriptive system comprises five variables: mobility, self-care, usual activities, pain/discomfort and anxiety/depression. Each dimension has three degrees: no problems, some problems, and extreme problems. The patient is asked to indicate health state by ticking the box next to the most appropriate statement in each of the five variables. This decision results into a number expressing the level selected for that dimension. The digits for the five variables can be combined into a 5-digit number describing the patient’s health state.

Our results demonstrated a correlation of ISS (rather than AIS) with EQOL.5D3L and it emerged as a significant prognostic factor for quality of life in patients with thoracic trauma. Since major thoracic trauma is very often part of a polytraumatic event, the ISS probably better identifies every single aspect of the complex event which will condition long-term quality of life in surviving patients. In our experience, in fact, 21 patients (75%) had concurrent extrathoracic trauma with orthopedic involvement in 13 patients (46.4%), central nervous system involvement in 6 patients (21.4%), visceral trauma in 6 patients (21.4%), vascular injuries in 4 patients (14.2%) while other regions were involved in 1 case (ocular trauma) (3.5%); moreover, the only case of in-hospital death was a polytraumatized patient with more than one extrathoracic region involvement.

Lung contusion is frequently observed in major thoracic trauma: in our experience, in fact, it was reported in more than half of the operated patients. Although lung contusions do not require any type of surgical treatment, they significantly affect the postoperative course. Lung contusion is defined as a pulmonary parenchyma injury without lung or vascular lacerations, resulting in defective gas exchange with arteriovenous shunting and reduced pulmonary compliance [21]. All these events may lead to respiratory failure, a life-threatening condition which may culminate in death.

In fact, following blood–air barrier damage, there is blood and interstitial fluid leakage into the alveolar and interstitial space, causing alveolar edema and surfactant reduction, culminating in alveolar collapse and atelectasis. As a consequence, impaired oxygenation causes vasoconstriction and altered perfusion, worsening both hypoxemia and hypercapnia [22]. The vast majority of lung contusions require only supportive treatment until damage healing, thus preventing respiratory failure.

Postural drainage, toilette bronchoscopy, incentive spirometry and coughing stimulation, and chest physiotherapy with deep breathing represent the cornerstones for preventing pulmonary atelectasis. In the ICU, prevention of lung derecruitment is achieved by applying continuous positive airway pressure (CPAP) using non-invasive and invasive techniques. Positive pressure ventilation can potentially add volume or excessive pressure damage and is therefore set with reduced tidal volumes calculated based on ideal body weight and with tight plateau pressure limits [23]. However, in the case of low thoraco-pulmonary compliance and/or pneumothorax or bronchopleural fistula, the ventilation strategy can become challenging. Moreover, even if non-invasive ventilation is applied, vigorous spontaneous respiratory efforts can potentially lead to patient self-inflicted lung injury. Additionally, in the case of unilateral lung contusion, the placement of the contused lung in a non-dependent position supports alveoli recruitment while prone positioning makes it possible to reduce pressure on the diaphragm [11,12,24].

We acknowledge the limits of this study: the small sample size and the retrospective monocentric nature of this study.

## 5. Conclusions

Major thoracic trauma represents a life-threatening condition, requiring prompt multidisciplinary approach and appropriate pathways for effective recovery. While acute morbidity and mortality are well-known outcomes in thoracic-traumatized patients, long-term quality of life in patients surviving surgical treatment has not been widely investigated before. We observed that ISS—rather than AIS—is correlated with the EQOL.5D3L questionnaire on long-term quality of life and represents the best prognostic factor, in terms of long-term quality of life—in patients surviving major thoracic trauma surgical treatment.

## Figures and Tables

**Table 1:** Demographics, trauma dynamics, surgical procedures, follow up. Qualitative variables were expressed in absolute value (percentage), while quantitative variables were expressed in the form of median (range).

Variables	
**Demographics**	
Age, years	60.3 (24–80)
Male gender	17 (60.7%)
Comorbidities	15 (50.3%)
Major	7 (28.5%)
Minor	8 (25%)
Fractured ribs	9.5 (2–10)
Sternal fracture	6 (21.4%)
Flail chest	21 (75%)
Bilateral thoracic trauma	18 (64.2%)
Lung contusion	15 (53.5%)
Concurrent extrathoracic trauma	21 (75%)
ISS	21 (4–75)
AIS	3 (2–4)
OIS	2 (1–5)
**Trauma Dynamics**	
Vehicle vs. vehicle	7 (25%)
Vehicle vs. non-vehicle	9 (32.1%)
Fall > 2 m	2 (7.1%)
Fall < 2 m	5 (17.8%)
Other	5 (17.8%)
**Surgical procedures**	
Plates and screws	24 (85.7%)
Stitches	1 (3.5%)
Combined/other	3 (10.7%)
Repaired ribs	3.46 (1–8)
Sternal stabilization	2 (7.1%)
Duration, minutes	153.67 (60–315)
Blood Transfusion	16 (57.1%)
Total LOS, days	17 (2–86)
ICU LOS, days	8 (0–43)
Postoperative pneumonia	9 (32.1%)
Other complications	5 (17.8%)
In-hospital mortality	1 (3.5%)
**Follow up**	
EQ-5D-3L scale quality of life	0.857 (0.027–1)
Dyspnea at rest	0
Exertional dyspnea	8 (28.5%)
Numerical Rate Pain Score (NRPS)	2 (1–6)
Long-term mortality	1 (3.5%)

ISS: Injury Severity Score; AIS: Abbreviated Injury Scale; OIS: Organ Injury Scale; LOS: Length of Stay; ICU: Intensive Care Unit.

## Data Availability

All data are available on request.
